# Dexmedetomidine and the glymphatic system: a new perspective in managing postoperative cognitive dysfunction

**DOI:** 10.3389/fphar.2025.1648308

**Published:** 2025-08-01

**Authors:** Sha-Sha Zhang, Fan-Geng Meng, Yuan-Yuan Rong, Yi-Wen Zhang, Hua-Qin Liu

**Affiliations:** ^1^ Department of Anesthesiology, The Fourth Hospital of Hebei Medical University, Shijiazhuang, Hebei, China; ^2^ Department of General internal medicine, Hebei Province Hospital of Chinese Medicine, Shijiazhuang, Hebei, China

**Keywords:** dexmedetomidine, glymphatic system, postoperative cognitive dysfunction, aquaporin-4, noradrenergic system, neuroinflammation

## Abstract

Postoperative cognitive dysfunction (POCD) is a common and significant neurological complication, occurring more frequently in elderly individuals and those with frailty or underlying neurodegenerative conditions, though it is not limited to these populations. The glymphatic system—a brain-wide clearance network dependent on aquaporin-4 (AQP4) polarity, arterial pulsation, and sleep-driven cerebrospinal fluid (CSF)–interstitial fluid exchange—has recently emerged as a promising target for cognitive protection. Dexmedetomidine (Dex), a selective α2-adrenergic receptor agonist, facilitates glymphatic function by mimicking non-REM sleep patterns and reducing central norepinephrine tone. Preclinical studies suggest Dex enhances glymphatic clearance by promoting CSF flow, restoring AQP4 localization, and attenuating neuroinflammation, potentially reducing POCD risk. Additionally, Dex provides neuroprotection by inhibiting neuronal apoptosis and preserving blood-brain barrier integrity. Despite promising evidence, most current data are derived from animal studies, and direct clinical validation remains limited. Key challenges include inadequate clinical tools for assessing glymphatic function and the absence of standardized protocols regarding Dex dosage, timing, and patient selection. This review provides a comprehensive summary of how Dex modulates glymphatic system function, with a particular focus on its potential to prevent POCD through mechanisms such as promoting CSF flow, restoring AQP4 polarity, and attenuating neuroinflammation. It also highlights current research gaps, including the lack of direct clinical evidence, the limited availability of reliable methods to assess glymphatic function, and the absence of standardized Dex administration protocols. The review emphasizes the need for future studies to incorporate multimodal imaging, integrated mechanistic analysis, and identification of high-risk patient subgroups, in order to facilitate the clinical translation of Dex as a glymphatic-targeted neuroprotective agent.

## 1 Introduction

Postoperative cognitive dysfunction (POCD) is a common and clinically significant neurological complication in the perioperative period, occurring more frequently in elderly individuals and those with frailty or underlying neurodegenerative conditions, though it is not limited to these populations (Bhushan et al., 2021; Peden et al., 2021; Deiner and Silverstein, 2009; Moller et al., 1998; Figueiredo and Devezas, 2024; O'Gara et al., 2021; Evered et al., 2018; Berger et al., 2018; Jin et al., 2020). Clinically, POCD is characterized by altered mental status, impaired environmental awareness, attention deficits, perceptual disturbances (e.g., hallucinations), and cognitive dysfunction, including disorientation and transient memory loss (Deiner and Silverstein, 2009; Suraarunsumrit et al., 2024). These symptoms can substantially hinder postoperative recovery and negatively impact long-term quality of life.

Although the precise pathophysiology of POCD remains elusive, current evidence suggests that it involves a constellation of interrelated mechanisms, including neuroinflammation, oxidative stress, impaired cerebral perfusion, and inadequate clearance of metabolic waste products from the brain (Liu et al., 2023; Xu et al., 2023; Bonfante et al., 2024). While anti-inflammatory agents have shown promise in animal models, their efficacy in clinical settings remains limited. For instance, high-dose intraoperative dexamethasone failed to reduce POCD incidence after cardiac surgery (Ottens et al., 2014), and clinical trials on parecoxib are limited in number and primarily conducted in China (Daojun et al., 2025). Moreover, adverse effects—such as increased risk of postoperative bleeding, particularly gastrointestinal hemorrhage in elderly patients—further constrain their routine clinical application (Dirkmann et al., 2015).

The multifactorial nature of POCD was further underscored in a 2024 systematic review by Figueiredo and Devezas, which highlighted the heterogeneity of POCD phenotypes and their association with diverse mechanisms, including inflammation, cellular stress, neuronal injury, and genetic vulnerability (Figueiredo and Devezas, 2024). This complexity is further supported by the absence of universally accepted diagnostic biomarkers, indicating that POCD cannot be fully explained by inflammation alone. Therefore, anti-inflammatory therapy alone is likely insufficient (Yu et al., 2022), emphasizing the urgent need to explore novel mechanisms and therapeutic targets more closely aligned with the pathophysiology of human cognition (Huang et al., 2019; Huang et al., 2020; Wang et al., 2019).

Currently, evidence-based guidelines advocate for multimodal non-pharmacological strategies—such as orientation therapy, sleep optimization, and early mobilization—as foundational preventive approaches (Wildes et al., 2019; Aldecoa et al., 2024). Pharmacological options remain limited, with only a few agents—such as dexmedetomidine (Dex), melatonin, and sufentanil—supported by high-quality evidence for reducing POCD risk (Zeng et al., 2023; Zhang et al., 2025; Wang et al., 2022; Yu et al., 2022; Zhao et al., 2020). Recent studies suggest that Dex may exert neuroprotective effects by enhancing glymphatic circulation and promoting the clearance of neurotoxic metabolites from the brain (Wang et al., 2024). The glymphatic system, a recently identified waste clearance pathway in the central nervous system, has emerged as a potentially critical factor in cognitive dysfunction (Li et al., 2024). Its function relies on several key elements—including optimal sleep architecture, aquaporin-4 (AQP4) polarization, and cerebrovascular dynamics—all of which are commonly impaired in individuals at high risk for POCD, such as older adults or those with sleep disorders and chronic inflammation. Therefore, enhancing glymphatic activity may represent a novel and actionable therapeutic strategy for POCD prevention.

This review aims to synthesize current evidence on Dex-mediated enhancement of glymphatic function. It explores the underlying mechanisms—including improvements in cerebrospinal fluid (CSF) dynamics, restoration of AQP4 polarity, and suppression of neuroinflammation—and discusses their potential translational implications for clinical management of POCD.

## 2 Methods

This narrative review is not a meta-analysis. A literature search was conducted using PubMed, Embase, and Web of Science with keywords including “glymphatic system,” “dexmedetomidine,” “sleep,” “POCD,” “AQP4 polarization,” and “perioperative neurocognitive disorders.” Studies were selected based on their relevance to POCD, glymphatic function, and Dex pharmacology. Publications lacking empirical data or based primarily on opinion without substantial experimental or clinical evidence were excluded. No quantitative or statistical analyses were performed. Instead, the literature was qualitatively synthesized to provide an integrative understanding of glymphatic mechanisms, perioperative cognitive management, and pharmacological interventions for POCD.

## 3 The glymphatic system: structure and modulatory factors

### 3.1 Polarized AQP4 expression: the structural core of the glymphatic system

The glymphatic system, first clearly characterized by Nedergaard’s group in 2012, is a waste clearance pathway in the brain (Iliff et al., 2012). In this system, CSF enters the brain parenchyma through periarterial spaces and exchanges with interstitial fluid (ISF), enabling the clearance of metabolic wastes such as β-amyloid and tau via perivenous pathways into meningeal lymphatics and ultimately the deep cervical lymph nodes. Impaired glymphatic function has been closely associated with neurodegenerative diseases and the development of POCD (Fyfe, 2025).

Aquaporin-4 (AQP4), a water channel highly expressed in the perivascular endfeet of astrocytes, plays a pivotal structural role in facilitating CSF–ISF exchange (Iliff et al., 2012; Chen et al., 2025; Salman et al., 2022). Polarized AQP4 distribution is essential for maintaining convective fluid flow and efficient metabolic clearance within the brain. Regulation of AQP4 polarity is multifaceted, involving transcriptional control, post-translational modifications (e.g., phosphorylation), metal ions, small-molecule modulators, circadian rhythm, and various intracellular signaling pathways (Vandebroek and Yasui, 2020; Hablitz et al., 2020). Experimental studies have shown that the loss of AQP4 polarization significantly reduces CSF influx and impairs solute clearance (Kress et al., 2014; Hablitz et al., 2020). Although AQP4 knockout is non-lethal, it markedly disrupts glymphatic transport efficiency (Mestre et al., 2018). Loss of AQP4 polarity has been implicated in Alzheimer’s disease, post-stroke cognitive impairment, and POCD. Aging, chronic inflammation, and neurodegeneration can disrupt perivascular AQP4 localization, leading to neurotoxic protein accumulation (e.g., Aβ, phosphorylated tau), neuroinflammation, and cognitive decline. Clinically, impaired AQP4 polarization may represent a shared pathological feature in populations at high risk for POCD, including elderly individuals, those with chronic inflammation, or sleep disorders. Thus, AQP4 polarity is not only a key anatomical determinant of glymphatic efficacy but also a promising therapeutic target for mitigating cognitive impairment.

### 3.2 Sleep: a physiological window for glymphatic activation

Sleep—especially non-rapid eye movement slow-wave sleep—is a critical period for glymphatic clearance (Xie et al., 2013; O'Gara et al., 2021). During this phase, delta waves dominate the electroencephalogram (EEG), accompanied by reduced noradrenergic tone, astrocyte shrinkage, and expansion of the extracellular space (ECS). These changes facilitate enhanced CSF–ISF exchange and accelerate the clearance of neurotoxic metabolites such as Aβ and phosphorylated tau (Hauglund et al., 2025; Benveniste et al., 2019). Animal studies suggest that glymphatic efficiency increases by up to 90% during natural sleep or anesthesia, with ECS volume expanding by as much as 60% (Xie et al., 2013; Voumvourakis et al., 2023). Clinically, CSF levels of Aβ and tau peak before sleep and reach their lowest levels in the early morning, supporting the notion of enhanced clearance during sleep (Lucey et al., 2018). Advanced neuroimaging (e.g., DTI-ALPS index) has revealed impaired glymphatic function and reduced CSF clearance in individuals with sleep disturbances. These alterations correlate with disrupted structure–function coupling and cognitive deficits. In contrast, enhanced structure–function coupling and ALPS indices are associated with preserved cognition in well-rested individuals.

These findings suggest a pathological feedback loop linking sleep impairment to cognitive dysfunction: sleep deprivation leads to AQP4 depolarization, which impairs glymphatic clearance, resulting in Aβ/p-tau accumulation, neuroinflammation, synaptic dysfunction, and ultimately cognitive decline. This cascade may underlie both POCD and neurodegenerative disorders such as Alzheimer’s disease. In summary, sleep serves as a critical window for brain waste clearance. Disruption of sleep quality or architecture may be a key contributor to POCD pathogenesis.

### 3.3 Noradrenergic tone and EEG rhythms: neuromodulatory drivers of glymphatic function

The noradrenergic system, primarily driven by the locus coeruleus, regulates arousal states and astrocytic physiology, thus influencing glymphatic function. During the waking state, elevated norepinephrine (NE) levels induce astrocytic swelling and reduce ECS volume, impeding CSF–ISF exchange (Bordon, 2024). In contrast, NE levels decline during non-rapid eye movement sleep or anesthesia, facilitating astrocyte relaxation, ECS expansion, and glymphatic flow (Persson et al., 2022; Hauglund et al., 2025). This state corresponds to slow-wave EEG activity, a hallmark of glymphatic activation. Melatonin, a circadian hormone secreted at night, promotes slow-wave sleep, reduces central NE levels, supports ECS expansion, and facilitates glymphatic clearance. Additionally, it exerts anti-inflammatory and antioxidant effects, helping to preserve AQP4 polarity and astrocyte integrity. Together, these findings indicate that both endogenous and pharmacological modulation of noradrenergic tone—especially through interventions that enhance slow-wave sleep—may offer effective strategies to support glymphatic clearance and reduce POCD risk, particularly in vulnerable populations.

### 3.4 Other mechanophysical regulators: Hemodynamic and positional modulators of glymphatic efficiency

Beyond molecular pathways, several mechanical and physiological forces modulate glymphatic function. Arterial pulsation, driven by cardiac and respiratory rhythms, serves as a primary motive force for CSF influx along perivascular spaces (Iliff et al., 2013; Feinberg and Mark, 1987; Rasmussen et al., 2022). Reduced vascular compliance (e.g., in aging or atherosclerosis) disrupts this pulsatility and compromises waste clearance (Rasmussen et al., 2022). Cerebral hypoperfusion further reduces CSF flow and promotes metabolite accumulation, contributing to cognitive impairment. Additionally, intracranial pressure fluctuations and body posture influence CSF–ISF exchange, highlighting the importance of systemic and positional factors in glymphatic transport. Recent research indicates that synchronized delta oscillations during sleep enhance CSF dynamics (Hablitz et al., 2019). Emerging non-invasive interventions—such as gamma-frequency entrainment and low-intensity ultrasound—have shown potential to modulate glymphatic flow (Murdock et al., 2024; Xie et al., 2024). Collectively, these mechanophysical forces interact with molecular regulators to shape glymphatic efficiency under both physiological and pathological conditions.

## 4 Dexmedetomidine and glymphatic modulation: preclinical evidence

### 4.1 Pharmacological properties of dexmedetomidine

**FIGURE 1:**
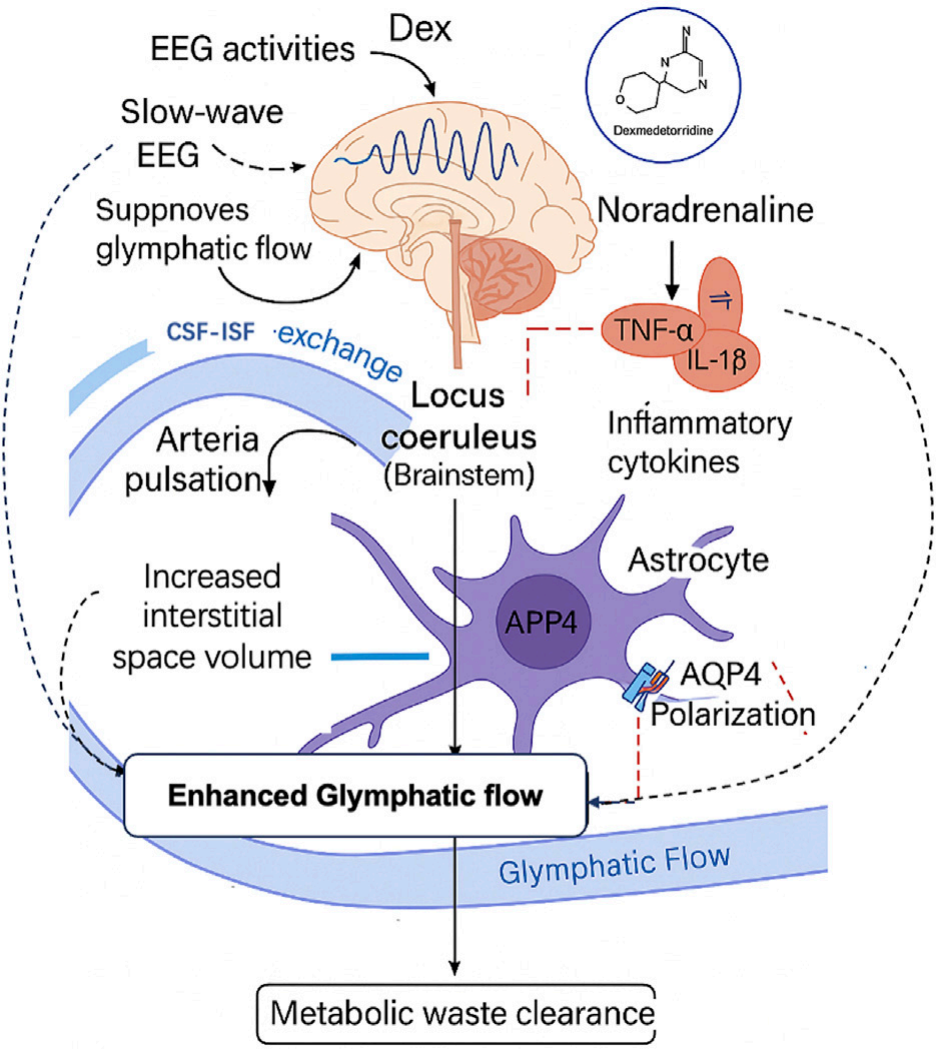
Dexmedetomidine promotes glymphatic function through neural, inflammatory, and vascular pathways to reduce postoperative cognitive dysfunction. Proposed mechanisms by which dexmedetomidine enhances glymphatic function and mitigates postoperative cognitive dysfunction (POCD). Dex suppresses locus coeruleus–mediated noradrenaline release, induces slow-wave EEG activity, reduces neuroinflammation, and restores AQP4 polarization in astrocytes. These changes facilitate cerebrospinal fluid (CSF) exchange, increase glymphatic flow, and promote clearance of neurotoxic metabolites.

Dex is a highly selective α2-adrenergic receptor agonist widely used in clinical anesthesia for its sedative, analgesic, and anxiolytic properties, as well as its ability to improve perioperative recovery (Verret et al., 2024; Arcangeli et al., 2009; Tan and Ho, 2010). Dex primarily exerts its sedative effects through inhibition of NE release in the locus coeruleus, producing a state that closely resembles physiological non-REM sleep without causing significant respiratory depression (Huupponen et al., 2008). In addition to its sedative properties, Dex exhibits potent anti-inflammatory effects. It has been shown to reduce levels of proinflammatory cytokines such as tumor necrosis factor-alpha (TNF-α), interleukin-1β (IL-1β), and interleukin-6 (IL-6), thereby attenuating both systemic and neuroinflammation induced by surgical trauma or ischemia–reperfusion injury (Wang et al., 2022; Li et al., 2015; Bao and Tang, 2020). Dex also confers neuroprotection by inhibiting neuronal apoptosis, preserving the blood–brain barrier, and reducing ischemic or surgery-related brain injury (Cai et al., 2025; Gao et al., 2020; Li et al., 2008; Zhai et al., 2019; Zhu et al., 2019). Together, these multifaceted pharmacological actions make Dex a promising candidate for preventing POCD, particularly via mechanisms involving glymphatic enhancement (Figure 1).

### 4.2 Summary of key studies on glymphatic modulation

Preclinical studies in recent years have provided compelling evidence that Dex enhances glymphatic function (Table 1). In a mouse model, Persson et al. reported that ketamine/dexmedetomidine (K/Dex) significantly increased CSF influx and improved metabolic waste clearance efficiency (Persson et al., 2024). Additional studies have demonstrated that Dex restores polarized AQP4 localization in astrocytic endfeet, facilitating CSF–ISF exchange and glymphatic transport (Wang et al., 2024; Xie et al., 2021). In models of lipopolysaccharide (LPS)-induced neuroinflammation, Dex not only mitigated neuroinflammation but also improved glymphatic clearance capacity (Zeng et al., 2021). Mechanistically, Dex mimics natural non-rapid eye movement sleep-like EEG activity and suppresses central noradrenergic tone, creating an optimal environment for glymphatic activation (Persson et al., 2022). Imaging studies using MRI and diffusion tensor imaging–analysis along the perivascular space (DTI–ALPS) further support these findings (Hsu et al., 2023). After Dex administration, increased CSF flow and improved structural integrity of glymphatic pathways were observed (Persson et al., 2022). While indirect, these imaging-based results strengthen the rationale for further mechanistic and clinical investigations. Additionally, Dex has demonstrated neuroprotective effects in models of stroke, hemorrhage, and traumatic brain injury, where it reduces neuronal apoptosis, suppresses inflammation, and improves behavioral outcomes. These findings support the hypothesis that Dex enhances glymphatic activity as part of its neuroprotective repertoire.

**TABLE 1:** Summary of animal studies evaluating the effects of dexmedetomidine on glymphatic function.

Author (year)	Animal model	Dex dose & route	Mechanistic focus	Glymphatic outcome	Conclusion
Wang et al. (2024)	Young mice (sevoflurane-induced glymphatic dysfunction)	25 μg/kg, i.p.	PI3K/AKT/ΔFosB/AQP4	↑ CSF flow, ↑ AQP4 polarity	Dex restored glymphatic circulation via PI3K/AKT/AQP4.
Wang S. et al. (2024)	C57BL/6 mice under sevoflurane anesthesia	25 μg/kg, i.p.	AQP4 depolarization, CSF flow	↑ glymphatic perfusion, ↓ ΔFosB	Dex reversed anesthesia-induced AQP4 mislocalization.
Xie et al. (2021)	Aged mice (laparotomy-induced POCD)	30 μg/kg, i.p.	Microglia, AQP4, synapse integrity	↓ inflammation, ↑ AQP4, ↑ cognition	Dex preserved AQP4 and reduced POCD in aged mice.
Zeng et al. (2021)	LPS-treated hippocampal neurons	1 μM, *in vitro*	AKT/GSK-3β/CRMP2, AQP4	↑ CRMP2, ↑ neuronal viability, ↑ AQP4	Dex activated CRMP2 signaling, improved AQP4 integrity.
Persson et al. (2024)	Mechanically ventilated rats	15 μg/kg/h, i.v.	CSF responsiveness to CO_2_, glymphatic flux	↑ glymphatic clearance under Dex	Dex reduced CO_2_-linked glymphatic reactivity.
Chen et al. (2025)	Mice with collagenase-induced ICH	30 μg/kg, i.p. × 3 days	AQP4 activation, CSF tracer clearance	↑ CSF tracer influx, ↑ AQP4 polarization	Dex restored post-ICH AQP4-dependent clearance.
Zhu et al. (2019)	LPS-induced neuroinflammatory mouse model	25 μg/kg, i.p.	JNK pathway, inflammation, AQP4	↓ neuroinflammation, ↑ AQP4 integrity	Dex modulated JNK to preserve AQP4 and glymphatic flow.
Zhai et al. (2019)	Mice with focal cerebral ischemia	20 μg/kg, i.p. × 3 days	σ1R-mediated AQP4 polarization	↑ AQP4 localization, ↓ infarct volume	Dex activated σ1R to restore AQP4 and protect brain tissue.

This table summarizes preclinical studies that investigated how dexmedetomidine (Dex) modulates glymphatic function through various pathways. Included models span sevoflurane-induced glymphatic dysfunction, postoperative cognitive impairment, and inflammatory injury. Most studies demonstrate that Dex enhances glymphatic transport by restoring AQP4 polarity, reducing neuroinflammatory signaling, or improving cerebrospinal fluid dynamics.

Although direct clinical studies on Dex and glymphatic function are still lacking, systematic reviews and meta-analyses have identified Dex as a potentially effective agent for POCD prevention. Future studies incorporating dynamic CSF imaging, ALPS index quantification, and biomarker monitoring—combined with large-scale prospective trials—will be crucial to establishing a definitive link between Dex and glymphatic function in humans.

### 4.3 Overview of mechanistic diversity and therapeutic implications

A growing body of preclinical research indicates that Dex exerts glymphatic-supportive effects via multiple converging mechanisms. These include enhancement of CSF–ISF exchange, restoration of AQP4 polarity, suppression of neuroinflammation, and improvement of cognitive outcomes. Notably, the effects of Dex appear dose- and time-dependent (Yu et al., 2022). Several signaling pathways have been implicated in Dex-mediated glymphatic regulation. These include the PI3K/AKT/ΔFosB axis, the σ1 receptor pathway, and the AKT/GSK-3β/CRMP2 cascade, all of which influence AQP4 localization and astrocytic function. Moreover, Dex-induced suppression of locus coeruleus activity decreases NE release, facilitating astrocytic relaxation and expansion of interstitial space—both essential for efficient glymphatic flow and slow-wave EEG activity. Animal models employed in these studies range from LPS-triggered neuroinflammation and sevoflurane-induced glymphatic dysfunction to POCD simulation. Despite heterogeneity in model design, Dex dosage (typically 15–50 μg/kg), and route of administration (intraperitoneal or intravenous), results consistently demonstrate Dex-enhanced glymphatic flow and neuroprotection. Collectively, these findings suggest that Dex promotes glymphatic clearance through both structural restoration and functional modulation. These preclinical insights provide a robust foundation for future clinical research into Dex as a glymphatic-targeted perioperative neuroprotective agent.

## 5 Mechanisms of dexmedetomidine in preventing POCD

### 5.1 Modulation of slow-wave EEG activity to facilitate glymphatic clearance

Dex induces a sedative state that closely mimics non-rapid eye movement sleep, characterized by increased slow-wave EEG activity (0.5–4 Hz) (Nelson et al., 2003). These slow-wave oscillations are strongly associated with enhanced CSF–ISF exchange and are regarded as the electrophysiological basis for active glymphatic clearance. In contrast, high-frequency EEG patterns during the waking state (e.g., beta and gamma waves) are linked to suppressed glymphatic transport. By inhibiting NE release from the locus coeruleus and reducing central excitability, Dex enhances slow-wave activity, optimizes the cerebral microenvironment, and promotes glymphatic function.

### 5.2 Restoration of AQP4 polarity in astrocytes

Dex may help restore the polarized expression of AQP4water channels in astrocytic endfeet. In animal models of aging or brain injury, Dex has been shown to reestablish AQP4 polarity, thereby enhancing perivascular CSF transport and improving the clearance of neurotoxic solutes (Wang et al., 2024). This mechanism is particularly relevant because AQP4 depolarization is associated with glymphatic dysfunction and is implicated in neurodegenerative diseases such as Alzheimer’s disease, which shares pathophysiological features with POCD (Rasmussen et al., 2018; Peng et al., 2023).

### 5.3 Suppression of neuroinflammation and structural preservation

Surgical trauma and anesthetic exposure can elicit systemic inflammatory cascades that extend to the central nervous system, resulting in neuroinflammation, blood–brain barrier disruption, and impaired glymphatic transport. These processes promote microglial and astrocytic activation, increase the permeability of the blood–brain barrier, and lead to depolarization of AQP4, all of which compromise the convective clearance of interstitial waste. Dysregulated cytokine release, including elevated levels of tumor necrosis factor-alpha (TNF-α), interleukin-1β (IL-1β), and interleukin-6 (IL-6), exacerbates neuronal injury and cognitive impairment. Dex has been shown to exert potent anti-inflammatory effects by inhibiting the nuclear factor-kappa B (NF-κB) pathway and reducing the production of these proinflammatory cytokines (Hu et al., 2022). Furthermore, Dex attenuates the activation of microglia and astrocytes, thereby dampening glial-driven neurotoxicity and preserving the quiescent astrocytic phenotype necessary for glymphatic function. By maintaining AQP4 polarization and reducing blood–brain barrier permeability, Dex helps preserve the structural integrity of the glymphatic pathway, supporting efficient cerebrospinal fluid–interstitial fluid exchange. In animal models of surgical stress and neuroinflammation, Dex treatment resulted in reduced perivascular inflammatory infiltration, stabilized blood–brain barrier tight junction proteins such as claudin-5 and occludin, and prevented dislocation of AQP4 from astrocytic endfeet. These effects are critical for sustaining glymphatic transport under perioperative stress. Collectively, these findings suggest that the anti-inflammatory and structural-preserving properties of Dex constitute a central mechanism for its neuroprotective actions and highlight its potential role in mitigating POCD via glymphatic restoration.

### 5.4 Comparative advantages over other pharmacological strategies

Melatonin and Dex share several glymphatic-enhancing properties. Both agents promote slow-wave sleep, reduce central NE tone, and exhibit anti-inflammatory and antioxidant activities. These shared effects expand interstitial space and enhance CSF–ISF exchange, suggesting that both modulate glymphatic clearance via sleep-related mechanisms. However, Dex offers distinct advantages in the perioperative setting. As a potent α2-adrenergic agonist, Dex provides well-defined sedative, anxiolytic, and analgesic effects, and more directly suppresses locus coeruleus activity and NE release. In preclinical studies, Dex more consistently restores AQP4 polarity and enhances glymphatic CSF influx across various pathological conditions. Sufentanil, a synthetic opioid frequently used for perioperative analgesia, has been reported to reduce POCD incidence, likely through its analgesic and sedative effects. However, its impact on glymphatic function remains unclear. Unlike Dex or melatonin, sufentanil does not target noradrenergic signaling or astrocytic physiology and may disrupt sleep architecture, depending on dose and administration route. In summary, while melatonin and sufentanil offer partial neuroprotective effects, Dex uniquely combines sedative potency, central noradrenergic suppression, and glymphatic facilitation. These integrated actions make Dex a promising pharmacological candidate for POCD prevention through brain clearance enhancement.

## 6 Discussion

Dex, a widely used perioperative sedative, has been shown in multiple high-quality clinical trials to reduce the risk of POCD. However, its precise neuroprotective mechanisms remain incompletely understood, which limits the development of mechanism-based clinical applications. Emerging preclinical evidence suggests that, beyond its well-established anti-inflammatory, antioxidant, and anti-apoptotic effects, Dex may also enhance glymphatic function by promoting CSF flow and metabolic waste clearance. This mechanism aligns with the pathophysiological context of surgery-induced metabolic disturbances and age-related cognitive vulnerability. Several animal studies have demonstrated that Dex can restore AQP4polarity, suppress central noradrenergic tone, and increase slow-wave EEG activity—key features associated with enhanced glymphatic clearance. However, it is important to note that these findings are largely based on animal models, and direct clinical validation is still lacking (Sun et al., 2024; Qian et al., 2015).

There are several challenges in elucidating the relationship between Dex and glymphatic function. First, reliable and repeatable clinical tools for directly assessing glymphatic activity are currently limited. Although MRI-based diffusion tensor imaging along perivascular spaces (DTI–ALPS index) has been used as an indirect proxy, its sensitivity and specificity are suboptimal (Li et al., 2024). Second, Dex-related studies vary widely in dosing regimens, routes of administration, and timing, making cross-study comparisons difficult and limiting translational consistency. Moreover, current research primarily focuses on AQP4 expression and inflammatory pathways, while glymphatic regulation is influenced by a broader range of factors—including sleep architecture, cerebral perfusion, arterial pulsatility, neuronal oscillations, and even external neuromodulation (e.g., gamma-frequency entrainment). Whether Dex-induced slow-wave activity represents the central mechanism for glymphatic enhancement remains to be determined. Although Dex has shown promising neuroprotective effects for POCD prevention in non-cardiac surgery populations, its efficacy in cardiac surgery remains uncertain (Patel et al., 2022; Zhuang et al., 2024). This discrepancy may reflect differences in pathophysiological mechanisms across surgical types or patient profiles, highlighting the need for stratified studies. Furthermore, patients at highest risk for POCD—such as the elderly or those with diminished cognitive reserve—may already have impaired glymphatic function, making them both vulnerable and potentially responsive to glymphatic-modulating interventions. Evaluating Dex’s effects in these cognitive risk subgroups could guide more personalized, precision-based perioperative care.

Despite its generally favorable safety profile, Dex is not without limitations. As a potent α2-adrenergic receptor agonist, it can induce dose-dependent hypotension and bradycardia, particularly in older adults or those with pre-existing cardiovascular conditions. Over-sedation and respiratory depression may also occur, especially when used concomitantly with other sedatives. Therefore, Dex should be administered at the lowest effective dose with careful titration, and patients should be closely monitored for hemodynamic and respiratory changes. Caution is also warranted in populations such as patients with severe hepatic or renal impairment, pregnant or lactating women, and individuals with significant cardiac disease.

In conclusion, the hypothesis that Dex mitigates POCD by enhancing glymphatic clearance offers a novel and promising perspective for understanding POCD pathogenesis and developing effective interventions. However, this concept is still in its early translational stages. Future research should prioritize: (1) developing sensitive and reliable imaging methods for glymphatic assessment; (2) defining dose-, time-, and route-dependent effects of Dex; (3) elucidating its multimodal mechanisms through integrated analysis of EEG activity, perfusion, and inflammation; and (4) conducting stratified clinical studies based on patients’ baseline cognitive status. These efforts are crucial for advancing Dex as a glymphatic-targeting neuroprotective agent and enabling precision prevention of POCD.

## 7 Conclusion

Dex, beyond its established anti-inflammatory and neuroprotective properties, may mitigate POCD through enhancement of glymphatic clearance. Preclinical studies have highlighted its multifaceted effects, including the restoration of AQP4polarity, modulation of EEG slow-wave activity, and promotion of CSF dynamics. Despite these promising findings, clinical evidence remains limited. Future research should prioritize the development of advanced imaging tools, optimization of dosing strategies, and identification of high-risk populations to facilitate the effective clinical translation of glymphatic-targeted neuroprotective interventions.
